# Supplementary material for: Worldwide Surveillance Actions and Initiatives of Drinking Water Quality: A Scoping Review
Source: Int J Environ Res Public Health. 2022 Dec 29;20(1):559. doi: 10.3390/ijerph20010559 (PMC9819457; doi:10.3390/ijerph20010559)
Supplement: Supplementary file 1 [file ijerph-20-00559-s001.zip › ijerph-2097056-supplementary.pdf]

**Table S1.** Characterization of sample according to reference, graduation from the 1st author, country and objective of the study.

| Reference | Graduation from the 1st author | Country | Objective |
|---|---|---|---|
| 1. Jones, M. C. et al. (2020) 'Arsenic Concentrations in Ground and Surface Waters across Arizona Including Native Lands', Journal of Contemporary Water Research & Education, 169(1), pp. 44–60. doi: 10.1111/j.1936-704X.2020.03331.x. | Nursing | United States | To combine water quality and arsenic concentration data on the water in Arizona from various databases and scientists into a single location. |
| 2. Gunnarsdottir, M. J., Gardarsson, S. M., Elliott, M., Sigmundsdottir, G., & Bartram, J. (2012). Benefits of Water Safety Plans: Microbiology, Compliance, and Public Health. Environmental Science \& Technology, 46(14), 7782–7789. https://doi.org/10.1021/es300372h | Civil and Construction Engineering | Iceland | To determine the impact of Water Safety Plan (WSP) implementation on (a) regulatory compliance, (b) Microbiological determination of water, and (c) incidence of clinical diarrhea cases, using comprehensive surveillance data. |
| 3. Monti MM, David F, Shin M, Vaidyanathan A. Community drinking water data on the National Environmental Public Health Tracking Network: a surveillance summary of data from 2000 to 2010. Environ Monit Assess. 2019;191(9):557. | Biological Sciences | United States | To identify the degree to which ten drinking water contaminants are present in finished water delivered to populations served by community water systems (CWS) in 24 states from 2000 to 2010. |
| 4. Setty KE, Kayser GL, Bowling M, et al. Water quality, compliance, and health outcomes among utilities implementing Water Safety Plans in France and Spain. Int J Hyg Environ Health. 2017;220(3):513-530. | Environmental Health Scientist | France and Spain | To characterize changes in water quality, compliance, and gastrointestinal disease incidence following WSP (Water Safety Plan) implementation. |
| 5. Sinyange N, Brunkard JM, Kapata N, et al. Cholera Epidemic — Lusaka, Zambia, October 2017–May 2018. MMWR Morb Mortal Wkly Rep 2018;67:556–559. | Medicine | Zambia | Reporting a cholera outbreak that began in October 2017 in Zambia resulted in approximately 5,900 cases and 114 deaths. |
| 6. Yassin MM, Amr SS, Al-Najar HM. Assessment of microbiological water quality and its relation to human health in Gaza Governorate, Gaza Strip. Public Health. 2006;120(12):1177-1187. doi:10.1016/j.puhe.2006.07.026 | Biological Sciences | Palestin e | To assess the contamination level of total and fecal coliforms in water wells and distribution networks, and their association with human health in Gaza Governorate, Gaza Strip. |
| 7. Ochoo, Benjamin & Valcour, James. (2017). Association between perceptions of public drinking water quality | policy analyst | Canada | 1) Determine public perceptions of piped water quality and the perceived health risks, 2) assess the quality of public water supply (after |

| | | | |
|---|---|---|---|
| and actual drinking water quality: A community-based exploratory study in Newfoundland (Canada). Environmental research. 159. 435-443. 10.1016/j.envres.2017.08.019. | | | tratment of raw water treatment by local utilities), and 3) link public perception of the quality of piped water and the perceived health risks with the actual quality of piped water. |
| 8. Blackburn BG, Craun GF, Yoder JS, et al. Surveillance for waterborne-disease outbreaks associated with drinking water--United States, 2001-2002. MMWR Surveill Summ. 2004;53(8):23-45. | Medicine | United States | To present data for outbreaks associated with drinking water that occurred during 2001-2002. |
| 9. Ferretti E, Bonadonna L, Lucentini L, Della Libera S, Semproni M, Ottaviani M. A case study of sanitary survey on community drinking water supplies after a severe (post-Tsunami) flooding event. Ann Ist Super Sanita. 2010;46(3):236-241. doi:10.4415/ANN_10_03_03 | Chemistry | Sri Lanka | To support the identification of health priorities related to drinking water quality in three districts of Sri Lanka in order to provide information on health and social conditions of Tsunami affected populations and to improve microbiological and chemical water safety and surveillance measures in the flooded areas. |
| 10. Brunkard JM, Ailes E, Roberts VA, et al. Surveillance for waterborne disease outbreaks associated with drinking water---United States, 2007--2008. MMWR Surveill Summ. 2011;60(12):38-68. | Environmental Sciences | United States | To present data on 48 water-related outbreaks that occurred during 2007-2008 |
| 11. McCarty CL, Nelson L, Eitniear S, et al. Community Needs Assessment After Microcystin Toxin Contamination of a Municipal Water Supply - Lucas County, Ohio, September 2014. MMWR Morb Mortal Wkly Rep. 2016;65(35):925-929. Published 2016 Sep 9. doi:10.15585/mmwr.mm6535a1 | Bachelor of Science | United States | To present the communication strategies, water exposure and household needs derived from a "Community Assessment for Public Health Emergency Response (CASPER)" |
| 12. Abu Amr SS, Yassin MM. Microbial contamination of the drinking water distribution system and its impact on human health in Khan Yunis Governorate, Gaza Strip: seven years of monitoring (2000-2006). Public Health. 2008;122(11):1275-1283. doi:10.1016/j.puhe.2008.02.009 | Environmental engineering | Palestin e | To assess total and faecal coliform contamination in water wells and distribution networks over the past 7 years, and their association with human health in Khan Yunis Governorate, Gaza Strip. |
| 13. Liang JL, Dziuban EJ, Craun GF, et al. Surveillance for waterborne disease and outbreaks associated with drinking water and water not intended for drinking--United States, 2003-2004. MMWR Surveill Summ. 2006;55(12):31-65. | Biological Sciences Veterinary Medicine | United States | To present data on 36 water-associated outbreaks (WBDOs) that occurred during January 2003 to December 2004. |

| | | | |
|---|---|---|---|
| 14. Centers for Disease Control and Prevention (CDC). Epidemic typhoid fever--Dushanbe, Tajikistan, 1997. MMWR Morb Mortal Wkly Rep. 1998;47(36):752-756. | - | Tajikistan | To describe a major epidemic of typhoid fever in Dushanbe, Tajikistan, that resulted from contamination of the municipality water system. |
| 15. Aragonés Sanz N, Palacios Diez M, Avello de Miguel A, Gómez Rodríguez P, Martínez Cortés M, Rodríguez Bernabeu MJ. Nivel de arsénico en abastecimientos de agua de consumo de origen subterráneo en la comunidad de Madrid [Arsenic levels in drinking water supplies from underground sources in the community of Madrid]. Rev Esp Salud Publica. 2001;75(5):421-432. | Medicine | Spain | To determine the concentration of arsenic in water from underground sources in the Community of Madrid. |
| 16. Centers for Disease Control and Prevention (CDC). Assessment of inadequately filtered public drinking water--Washington, D.C., December 1993. MMWR Morb Mortal Wkly Rep. 1994;43(36):661-669. | - | United States | To describe results of 4 investigations to determine whether excessive cases of diarrheal illness occurred because residents drank improperly filtered water. |
| 17. Barwick RS, Levy DA, Craun GF, Beach MJ, Calderon RL. Surveillance for waterborne-disease outbreaks--United States, 1997-1998. MMWR CDC Surveill Summ. 2000;49(4):1-21. | Bachelor of Science | United States | To present data on 17 outbreaks associated with drinking water that occurred from 1997 to 1998. |
| 18. Levy DA, Bens MS, Craun GF, Calderon RL, Herwaldt BL. Surveillance for waterborne-disease outbreaks--United States, 1995-1996. MMWR CDC Surveill Summ. 1998;47(5):1-34. | Medicine | United States | To present data on 22 outbreaks associated with drinking water that occurred from 1995 to 1996. |
| 19. Lee SH, Levy DA, Craun GF, Beach MJ, Calderon RL. Surveillance for waterborne-disease outbreaks--United States, 1999-2000. MMWR Surveill Summ. 2002;51(8):1-47. | - | United States | To present data on 39 outbreaks associated with drinking water that occurred in the period 1999-2000. |
| 20. Penman, A. D., Brackin, B. T., & Embrey, R. (1997). Outbreak of acute fluoride poisoning caused by a fluoride overfeed, Mississippi, 1993. Public health reports (Washington, D.C. : 1974), 112(5), 403–409. | Medicine | United States | To determine the extent and confirm the cause of an August 1993 outbreak of acute fluoride poisoning in a small Mississippi community, thought to result from excess fluoride in the public water supply. |
| 21. Khare P. A large-scale investigation of the quality of groundwater in six major districts of Central India during the 2010-2011 sampling campaign. Environ Monit Assess. 2017;189(9):429. doi:10.1007/s10661-017-6130-0 | Civil Engineering | India | To investigate the groundwater quality in six major districts of Madhya Pradesh in central India,namely, Balaghat, Chhindwara, Dhar, Jhabua, Mandla,and Seoni during the 2010–2011 sampling campaign,and |

| | | | |
|---|---|---|---|
| | | | discusses improvements made in the supplied water quality between the years 2011 and 2017. |
| 22. Strosnider H, Kennedy C, Monti M, Yip F. Rural and Urban Differences in Air Quality, 2008-2012, and Community Drinking Water Quality, 2010-2015 - United States. MMWR Surveill Summ. 2017;66(13):1-10. Published 2017 Jun 23. doi:10.15585/mmwr.ss6613a1 | - | United States | To assess environmental factors that affect health by analyzing three air quality measures and one water quality measure for 26 states during 2010–2015. |
| 23. Mora-Alvarado, DA; Rivera-NAvarro, P; Acuña-Cubero, F et al. Drinking-water and sanitation in Educational Institutions in Costa Rica 2017. Tecnología en marcha Journal; 2019: Vol. 32 especial. Laboratorio Nacional de Aguas; Pág 5-16 , | Chemistry | Costa Rica | To estimate the coverage of drinking water and sanitation in Costa Rica's educational institutions in 2017, in order to establish the baseline and targets for the 2030 Agenda within the scope of the Sustainable Development Goals. |
| 24. Elliot AJ, Hughes HE, Astbury J, et al. The potential impact of media reporting in syndromic surveillance: an example using a possible Cryptosporidium exposure in North West England, August to September 2015. Euro Surveill. 2016;21(41):30368. | Biology | Englan d | To present a description of real-time monitoring of health care seeking behavior using syndromic surveillance during a Boil Water Warning (BWN) following detection of Cryptosporidium in mains water supplies to parts of North West England between 31 July and August 4, 2015. |
| 25. Queiroz AC, Cardoso LS, Heller L, Cairncross S. Integrating surveillance data on water-related diseases and drinking-water quality; action-research in a Brazilian municipality. J Water Health. 2015;13(4):1048-1054. | Nursing | Brazil | To throw light on the process of data integration, based on the experience of one of the municipalities in which we worked, the one with intermediate population size. |
| 26. Knobeloch L, Gorski P, Christenson M, Anderson H. Private drinking water quality in rural Wisconsin. J Environ Health. 2013;75(7):16-20. | - | United States | Summarize the water quality results of a government testing program and discuss the importance of ensuring a safe domestic drinking water supply. |
| 27. Edwards JE, Henderson SB, Struck S, Kosatsky T. Characteristics of small residential and commercial water systems that influence their likelihood of being on drinking water advisories in rural British Columbia, Canada: a cross-sectional study using administrative data. J Water Health. 2012;10(4):629-649. doi:10.2166/wh.2012.046 | - | Canada | To characterize water systems in counseling for microbiological threats and to identify variables associated with the status and duration of counseling. |
| 28. Sanders AP, Messier KP, Shehee M, Rudo K, Serre ML, Fry RC. Arsenic in North Carolina: public health implications. Environ Int. 2012;38(1):10-16. | Biomedical engineering | United States | Comprehensively examine statewide arsenic trends and identify areas of public health concern. |

| | | | |
|---|---|---|---|
| 29. Yoder J, Roberts V, Craun GF, et al. Surveillance for waterborne disease and outbreaks associated with drinking water and water not intended for drinking--United States, 2005-2006 [published correction appears in MMWR Surveill Summ. 2008 Oct 10;57(40):1105]. MMWR Surveill Summ. 2008;57(9):39-62. | Social service | United States | To present data on 28 outbreaks associated with drinking water that occurred between 2005 and 2006. |
| 30. Zietz BP, Lass J, Suchenwirth R. Assessment and management of tap water lead contamination in Lower Saxony, Germany. Int J Environ Health Res. 2007;17(6):407-418. | - | Germa ny | To assess the present state of drinking water contamination with lead in Lower Saxony and to promote replacement of lead pipes. |
| 31. Tabbot, Peter N., and Mark G. Robson. "The New Jersey residential well-testing program--a case study: Randolph Township." Journal of Environmental Health 69, no. 2 (2006): 15+ | Ecology | United States | To present the results of the Randolph County Private Well Survey. |
| 32. Schuster CJ, Ellis AG, Robertson WJ, et al. Infectious disease outbreaks related to drinking water in Canada, 1974-2001. Can J Public Health. 2005;96(4):254-258. | Geography | Canada | To present data on waterborne disease outbreaks in Canada in order to gain a better understanding of the impact of drinking water quality on public health and disease burden. |
| 33. Moore AC, Herwaldt BL, Craun GF, Calderon RL, Highsmith AK, Juranek DD. Surveillance for waterborne disease outbreaks--United States, 1991-1992. MMWR CDC Surveill Summ. 1993;42(5):1-22. | - | United States | To present data on 34 outbreaks associated with drinking water that occurred in the period 1991-1992. |
| 34. Carvalho, Ana Paula Mendes et al. A vigilância em saúde ambiental como resposta ao desastre do rompimento da barragem de rejeitos em Brumadinho. Saúde em Debate [online]. v. 44, n. spe2 [Acessado 26 Abril 2022] , pp. 364-376. | Nursing | Brazil | To describe the actions developed by the environmental health surveillance of the Minas Gerais State Health Department (SES-MG) in response to the rupture of the B1 dam in Brumadinho. |
| 35. Romani, C.D., Stancari, R.C.A., Nascentes, G.A.N. e Anversa, L. 2018. Fluoretação das águas de abastecimento público: 10 anos de monitoramento em 38 municípios do Centro-Oeste Paulista, São Paulo, Brasil. Vigilancia Sanitaria em Debate. 6, 4 (nov. 2018), 47-55. | Biomedicine | Brazil | To evaluate the concentration of fluoride in public water supply in the municipalities covered by the Bauru Sanitary Surveillance Group. |
| 36. Piorunneck, Cristiane Matsuo de Oliveira, Ditterich, Rafael Gomes, & Gomes, Eliane Carneiro. (2017). Heterocontrole da fluoretação nos municípios da Região | Dentistry | Brazil | To analyze the heterocontrol of fluoridation in municipalities with a population of over 50,000 inhabitants in the Metropolitan Region of Curitiba, Paraná, in the years 2014 and 2015. |

| | | | |
|---|---|---|---|
| Metropolitana de Curitiba nos anos de 2014 e 2015. Cadernos Saúde Coletiva, 25(4), 414-422. | | | |
| 37. Guzmán, Blanca Lisseth, Nava, Gerardo, & Díaz, Paula. (2015). La calidad del agua para consumo humano y su asociación con la morbimortalidad en Colombia, 2008-2012. Biomédica, 35(spe), 177-190. | Veterinary Medicine | Colom bia | To analyze the results of water quality surveillance by health authorities in the territories of Colombia, as well as the association between water quality, infant mortality and morbidity from acute diarrheal diseases, foodborne diseases and hepatitis A. |
| 38. Teixeira ISC, Peresi JTM, Silva SIL, Ribeiro AK, Graciano RAS, Povinelli RF, et al. Solução alternativa coletiva de abastecimento de água (SAC): avaliação da qualidade bacteriológica e da cloração. Rev Inst Adolfo Lutz. São Paulo, 2012; 71(3):514-9 | Biomedicine | Brazil | To evaluate the bacteriological quality and free residual chlorine content in water samples from SAC in municipalities located in the northwest of the State of São Paulo, from 2004 to 2010. |
| 39. Motter, Juliana,Moyses, Simone Tetu,França, Beatriz Helena Sottile,Carvalho, Max Luiz de,Moysés, Samuel Jorge (2011) Análise da concentração de flúor na água em Curitiba, Brasil: comparação entre técnicas. Rev Panam Salud Publica;29(2) 120-125,fev. 2011. | Dentistry | Brazil | To evaluate the adequacy of fluoride levels in public water supply in Curitiba, Paraná State, Brazil, according to the electrometric technique and SPADNS (heterocontrol). |
| 40. Queiroz, Josiane Teresinha Matos de, Heller, Léo e Silva, Sara Ramos daAnálise da correlação de ocorrência da doença diarreica aguda com a qualidade da água para consumo humano no município de Vitória-ES. Saúde e Sociedade [online]. 2009, v. 18, n. 3, pp. 479-489. | Civil Engineering | Brazil | To trace possible associations of the quality of water for human consumption distributed to the population by VIGIÁGUA and Control of water quality, with the occurrence of cases of diarrhea. |
| 41. PORTO, Suely Kikuchi Sato Soares. Controle e vigilância de trihalomethanos em água de abastecimento humano: o caso do desastre ambiental dos rios Pomba e Paraíba do Sul. 2008. 143 f. Dissertação (Mestrado em Saúde Pública) - Escola Nacional de Saúde Pública Sergio Arouca, Fundação Oswaldo Cruz, Rio de Janeiro, 2008. | Chemistry | Brazil | To evaluate the monitoring of trihalomethanes (THM's), in the actions of the Water Quality Surveillance Program for Human Consumption carried out in the State of Rio de Janeiro, especially after the environmental accident at the Cataguases de Papel Industry. |
| 42. Castro, Ana Maria Santos Messeder de; Câmara, Volney de M. Avaliação do programa de vigilância da qualidade da água para consumo humano em Salvador, estado da Bahia. Rev. baiana saúde pública ; 28(2): 212-226, jul.-dez.2004. | - | Brazil | To analyze data from the Water Quality Surveillance Program developed by the city of Salvador-BA. |

| | | | |
|---|---|---|---|
| 43. Mora Alvarado, Darner Adrián; Araya García, Alvaro. (2008). Estado del agua para consumo humano y saneamiento en costa rica al año 2007. Revista Costarricense de Salud Pública, 17(32), 16-34. | Chemistry | Costa Rica | To describe the quality of water for human consumption (ACH ), excrement disposal, and sewerage water in Costa Rica, as well as their coverage to the year 2007. |
| 44. Iyer V, Choudhury N, Azhar GS, Somvanshi B. Drinking Water Quality Surveillance in a Vulnerable Urban Ward of Ahmedabad. Health (Irvine Calif). 2014;6(11):1165-1171. doi:10.4236/health.2014.611143 | Medicine | India | To investigate the drinking water surveillance parameters in a vulnerable neighborhood of Ahmedabad. |
| 45. Costa, Fábio da. Qualidade físico-química e microbiológica da água consumida em Guaíba (RS) e sua implicação na saúde. Dissertação (mestrado em saúde coletiva), 2018. Universidade Federal do Rio Grande do Sul. Escola de Enfermagem. Programa de Pós-Graduação em Saúde Coletiva. | Veterinary Medicine | Brazil | To evaluate whether there is a relationship between the records of diarrhea, notified by the sentinel network of Guaíba/RS, with the consumption of water outside of potability standards, in the health of this population. |
| 46. Soares, Nívia Cristiane Ferreira Brandão. Vigilância da qualidade da água para consumo humano no município de são luís: análise de indicadores ambientais. Dissertação (mestrado), 2015. Universidade Federal Do Maranhão; Programa De Pós-Graduação Em Saúde E Ambiente | Nursing | Brazil | To analyze environmental health indicators in the municipality of São Luís, based on data from the National Environmental Health Surveillance Information System (SINVSA), and from the National Water Quality Surveillance Program for Human Consumption (VIGIAGUA). |
| 47. Pachá, Anna Stella Cysneiros.Qualidade da água para consumo humano na Paraíba: Sistemas de Informações para fins de vigilância e controle das doenças diarreicas agudas. Dissertação (mestrado), 2018. Universidade Federal Da Paraíba; Centro De Ciências Exatas E Da Natureza; Programa Regional de Pós-Graduação em Desenvolvimento e Meio Ambiente – PRODEMA | Nursing | Brazil | To evaluate a possible correlation between two Health Information Systems in order to identify the coherence between the quality of water available from Alternative Collective Supply Solutions - SAC and the occurrence of Acute Diarrheal Diseases - ADD, in the period from 2012 to 2016, in the cities from João Pessoa, Guarabira and Campina Grande/PB. |
| 48. Oliveira, Julimara de Souza Costa et al. Soluções individuais de abastecimento de água para consumo humano: questões para a vigilância em saúde ambiental. Cadernos Saúde Coletiva [online]. 2017, v. 25, n. 2 [Acessado 26 Abril 2022] , pp. 217-224. | Veterinary Medicine | Brazil | To analyze possible health hazards related to the characteristics of the Individual Alternative Solutions registered, integrating data related to the notification of ADD cases in urban and rural areas of a municipality located in the Zona da Mata of Minas Gerais. |
| 49. Vasconcelos, Cíntia Honório et al. Surveillance of the drinking water quality din the Legal Amazon: analysis of | Ecology | Brazil | To evaluate the degree of implementation of VIGIAGUA in the municipalities that compose the Legal Amazon region, as well as analyze |

| | | | |
|---|---|---|---|
| vulnerable areas. Cadernos Saúde Coletiva [online]. 2016, v. 24, n. 1 | | | the data on drinking water, and to evaluate vulnerable areas in order to subsidize and offer direct strategic action for the monitoring of environmental health in this region. |
